# Parthenolide targets NF-κB (P50) to inhibit HIF-1α-mediated metabolic reprogramming of HCC

**DOI:** 10.18632/aging.204339

**Published:** 2022-10-18

**Authors:** Xiaorong Liu, Zhaofeng Gao, Xiaoguang Wang, Yiyu Shen

**Affiliations:** 1 Department of Hepatobiliary and Pancreatic Surgery, The Second Affiliated Hospital of Jiaxing University, Jiaxing 314099, Zhejiang, China

**Keywords:** parthenolide, hepatocellular carcinoma, NF-κB, HIF-1α, glycolysis

## Abstract

We focus on investigating the role of Parthenolide (Par), a small sesquiterpenoid molecule, in hepatocellular carcinoma (HCC) and its effective target.

Highly-metastatic HCC cells, MHCC97-H, were divided into the DMSO and the Par groups, of which the Par group was intervened at 5 and 10 mg/L doses. Cell viability was assessed by CCK-8 assay. Transwell chamber assay was performed to examine the metastatic and invasive abilities, while plate clone formation assay was conducted to detect the clone formation ability. For analysis of glucose uptake, glycolytic ability and lactate level, the glycolysis assay was employed. Brdu staining was performed to evaluate the cell proliferative potential. The P50 and HIF-1α levels were measured by immunofluorescence, while the expressions of p-P50 and HIF-1α were determined by Western-Blot. Small molecule–protein docking and Pull-down experiments were conducted to validate the Par–P50 binding model. After establishing the tumor-bearing mouse model, Par was administered by gavage to measure the tissue levels of P50 and HIF-1α, followed by plotting of tumor growth curves.

Par could inhibit the metastatic, invasive and clone formation abilities of MHCC97-H cells, reduce the cell proliferative potential, and suppress the glycolysis, as manifested by down-regulated level of lactate and reduced oxygen consumption. Meanwhile, Par inhibited the HIF-1α expression. We found that after silencing P50, the HIF-1α was down-regulated, the glycolytic ability decreased drastically, and the cellular metastatic and invasive abilities were suppressed. After P50 knockout, the effect of Par intervention on the MHCC97-H cells was reduced. In HCC-bearing mice, Par also exhibited an excellent anti-tumor effect, decreasing the tissue levels of P50 and HIF-1α.

This study discovers that Par can inhibit the HIF-1α-mediated glycolysis of HCC cells by targeting P50, thereby exerting an anti-tumor effect. P50 is a major effective target of Par.

## INTRODUCTION

Under sufficient oxygen, tumor cells still break down glucose through glycolysis to produce lactate. This phenomenon is referred to as the “Warburg effect”, which is also known as the “aerobic glycolysis” [1]. Tumor microenvironment hypoxia can activate glycolysis by suppressing oxidative phosphorylation and inhibit pyruvate catabolism and oxygen consumption [2]. Studies have demonstrated that HIF-1α can induce pyruvate dehydrogenase kinase 1 (PDK-1). By phosphorylating the E1 subunit of pyruvate dehydrogenase (PDH), PDK-1 further stimulates the PDH activity, resulting in the cytoplasmic accumulation of pyruvate [3]. HIF-1α can also increase the efficiency of glycolytic pathway by up-regulating the glucose transporters (GLUTs) and many other aerobic glycolysis-related genes [4]. Existing research has thus proven that HIF-1α is an important promoter of glycolysis. Since HIF-1α is also regulated by NF-κB transcription, it can be said that NF-κB-HIF-1α is the major regulatory signal of tumor glycolysis [5].

Parthenolide (Par), a small sesquiterpenoid molecule, has been found to possess a good inhibitory effect on multiple tumors. Its mechanism of action is associated with inducing apoptosis and enhancing tumor cytotoxicity [6]. However, its specific target requires further clarification. Through small molecule–protein docking, we found that Par might be a small regulatory molecule of P50. As an important subunit of NF-κB, P50 regulates the NF-κB activation. Hence, we further investigated the role of Par in a HCC model.

## MATERIALS AND METHODS

### Cell cultivation

After thawing, Highly-metastatic HCC cells, MHCC97-H cell lines (Procell Life Science and Technology, Wuhan, China) were cultured in an incubator (37°C, 5% CO_2_) with 10% FBS-containing DMEM. Cell viability was assessed with trypan blue reagent. The H22 cells were divided into the DMSO and Par groups after reaching logarithmic phase. The Par group was intervened at 5 and 10 mg/L doses. The DMSO group comprised control cells, which was treated with 0.1% DMSO.

We silenced P50 by RNAi technique, and transfected cells with siRNAs. The cells were divided into the DMSO and DMSO-RNAi groups for investigating the role of P50 in glycolysis. In further exploration of the correlation between P50 and Par, we divided the cells into the DMSO-RNAi and DMSO-RNAi+Par groups, where the intervention dose of Par was 10 mg/L.

### CCK-8

In cell viability assay, the H22 cells were seeded into 96-well plates, and 100 μl of complete DMEM was added to each well. After cells were adherent, the medium was replaced with Par-containing one, which was added at 100 μl per well. Then, 24 h later, 10 μl of CCK-8 reagent was added to each well for further incubation. The optical density (OD) value was measured 4 h later, and the cell viability was statistically analyzed against the blank medium control.

### Immunofluorescence

Immunofluorescence staining for P50 and HIF-1α was performed. H22 cells were cultured on coverslips, and intervened for 24 h with Par-containing medium after adherence. Then, the cells were washed thrice in pre-cooled PBS, fixed in 4% formaldehyde at room temperature for 0.5 h, and permeabilized with 0.2% Triton X-100 for 5 min, followed by overnight incubation at 4°C with P50 and HIF-1α monoclonal antibodies (1:500 dilutions; Abcam, MA, USA). Thereafter, the cells were washed twice in PBS, incubated with fluorescent secondary antibody, and then mounted with 95% glycerol and observed under a fluorescence microscope.

### Brdu

H22 cells were seeded into 12-well plates, and intervened for 24 h with Par-containing medium after adherence. Then, the cells were washed in PBS, added with Brdu reagent (concentration: 30 μM), and incubated for 1 h in the dark. After removing Brdu, the cells were washed once with PBS, treated with 1 ml of ethanol, and then incubated for 30 min with Triton X-100 at room temperature, followed by washing once with 1% BSA. Afterwards, 1-h incubation proceeded in the dark using 10 μl of anti-Brdu reagent mixed with 0.5% Tween-20 and 1% BSA, and a subsequent incubation with secondary antibody. Finally, the cells were nuclear-stained with DAPI, mounted with 95% glycerol and observed under a fluorescence microscope.

### Transwell

After liquefying Matrigel (BD Biosciences, NJ, USA) overnight for 24 h at 4°C, it was diluted at a 5:1 volume ratio in serum-free medium, mixed well and placed into the upper layer of Transwell chamber, followed by cultivation in a 37°C incubator for 4–5 h to allow solidification of gelatinous layer. The cells were cultured after transfection. The logarithmic cells were suspended in serum-free medium, and then seeded into the upper chamber, while the lower chamber was added with 20% FBS-containing complete medium. After a further 24-h incubation with Par-containing medium, the upper layer cells were removed from the Transwell chamber, washed twice with PBS, fixed in neutral formaldehyde, stained with 0.1% crystal violet solution and microscopically observed for the number of invaded cells. The operational procedure for metastasis assay was identical to the invasion assay, where the pretreatment with Matrigel was unnecessary.

### Plate clone formation

Logarithmic cells were collected from various groups, digested with 0.25% trypsin, pipetted into single cells, and suspended in 10% FBS-containing DMEM medium for subsequent use. The cell suspensions were diluted in multiples of gradient. Cells in various groups were separately seeded into a dish containing 37°C pre-warmed medium (10 mL) at a gradient density of 500 cells/dish, and gently rotated to allow uniform dispersion. This was followed by a 3-week cultivation of cells in a 37°C, 5% CO_2_ incubator with saturated humidity. The cultivation was terminated when macroscopically visible clones appeared in the petri dish. After discarding supernatant, the cells were washed carefully with PBS twice, and fixed in 5 mL of 4% paraformaldehyde for 15 min, followed by removal of the fixing solution. Transfection was performed with an appropriate amount of GIEMSA stain for 20 min, and then the staining solution was washed off slowly with running water, and the remaining was air-dried. Finally, clones were counted with the naked eye.

### Glycolysis assay [7]

(1) Determination of intracellular glucose uptake was achieved by measuring the cellular uptake of 3H-2-deoxyglucose (Sigma). Briefly, the cells were cultured in 12-well plates and pre-incubated in glucose-free medium for 30 min. Then, 3H-2-deoxyglucose was added at 1 μCi per well to the cells, followed by 30 min of incubation. After washing with PBS, the cells were lysed with 1% SDS. Liquid scintillation counting was performed for radioactivity measurement of the cell lysates, and the final step was normalization of the radioactivity to the lysate protein concentrations.

(2) For determination of intracellular glycolytic rate, the 5-3H-glucose conversion into 3H-H_2_O was monitored as formerly described. The first step was PBS washing of cells and a 30-min resuspension in glucose-free Krebs buffer (1 ml). Next, a 1-h resuspension of cells proceeded in Krebs buffer (0.5 ml) involving 5-[3H] glucose (5 mCi) and glucose (10 mM). Three aliquots, each 100 ml in volume, were shifted to PCR tubes (uncapped) containing 0.2 N HCl (100 ml), and each tube was shifted to a H_2_O (0.5 ml)-involving scintillation vial. This was followed by sealing of the scintillation vials and a 48-h reaction for diffusion purpose. Eventually, a liquid scintillation counter was utilized to assess the 3H diffusion, as well as the non-diffused 3H amount.

(3) For determination of intracellular lactate production, cells were subjected to cultivation in fresh media (absent of phenol red) and 12–24 h incubation prior to the medium harvesting. Biovision assay kits were utilized for examining the lactate production as per the protocol of manufacturer, followed by normalization of the lactate level against cell number.

### Western-blot

H22 cells were seeded into 6-well plates, and intervened for 24 h with Par-containing medium after adherence. All the cells were collected, washed twice with pre-cooled PBS, and lysed on ice for 30 min with NP-40 lysate (0.5 ml; Beyotime Biotechnology, Shanghai, China), followed by centrifugation for collecting total protein. Tumor tissues were ground with liquid nitrogen, and total protein was extracted with NP-40 lysate. Protein quantification was accomplished with BCA kit (Beyotime Biotechnology, Shanghai, China), and the protein concentration was adjusted. After SDS-PAGE gel electrophoresis and PVDF membrane transfer, the membranes were blocked for 2 h with 5% skimmed milk powder, and then incubated with p-P50 and HIF-1α monoclonal antibodies (TBST dilutions; Abcam, MA, USA). Following twice washing with TBST, a further incubation was carried out with HRP-labeled goat anti-rabbit IgG antibody (1:2000 dilution; Abcam, MA, USA). Finally, chemiluminescent immunoassay was performed, and OD was analyzed via Image Pro-Plus 6.0. The results were presented as OD comparisons between the target proteins and the internal reference (GAPDH).

### Establishment of mouse HCC model

Wild-type BALB/c mice were reared at the Animal Experiment Center of Jiaxing University. The murine experiments were approved by the Ethics Committee of Jiaxing University, which conformed to the animal ethics and welfare regulations. For establishment of HCC-bearing mouse model, the cultured H22 cells were collected at logarithmic phase, washed twice with PBS and density-adjusted to 5 × 10^5^/ml. Each nude mouse was injected with 0.2 ml of cell suspension into the foreleg armpit. Then, rearing continued in a clean environment for observing the growth of mice and the formation of solid tumors. On around 15^th^–18^th^ d, visible subcutaneous tumors appeared in mice. At this point, the mice were divided into the Control and Par groups. The Control group comprised naturally-grown tumor-bearing mouse. In the Par group, gavage administration was given at 5 and 10 mg/kg doses once daily for 15 consecutive d. The mice lived in a consistent environment, which were fed and watered ad libitum. Fifteen d later, the mice were killed by carbon dioxide asphyxiation and the tumor tissues were harvested.

### Immunohistochemical

Immunohistochemical (IHC) staining was performed to determine the P50 and HIF-1α levels. The tumor tissues were fixed in 4% paraformaldehyde, paraffin-embedded and sectioned. The resulting tissue sections were soaked in 1:50 acetone solution for 3 min, dried, and then treated with xylene and absolute ethanol. Following antigen retrieval in 0.01 mol/L citrate buffer at 92–98°C, the tumor tissue sections were treated with 3% hydrogen peroxide for 10–15 min to eliminate endogenous peroxidase, and then blocked with 5% BSA at 37°C for 15–30 min. Afterwards, the sections were incubated with Caspase-3 monoclonal antibody at 37°C for 1–2 h, and further with HRP-labeled avidin at 37°C for 20 min, followed by 3–5 min staining with DAB. Finally, the tissue sections were counterstained with hematoxylin, dehydrated, permeabilized and mounted with resin.

### H&E

Mouse tumor tissues were treated with formalin for 0.5 h, dehydrated, permeabilized, paraffin-embedded and sectioned. Tissue sections were baked at 45°C for 2 h, and then treated with gradient concentrations of xylene and ethanol. After washing with water, the tissue sections were stained with hematoxylin for 10 min, rinsed with tap water and then treated with 1% hydrochloric acid-alcohol, followed by ethanol dehydration and eosin staining. Finally, the tissue sections were dehydrated, permeabilized, mounted with neutral gum and observed under an optical microscope.

### Molecular docking and pull-down experiments

P50 was searched in the Protein Data Bank (PDB ID: 1SVC). By setting up appropriate box centers (center_x = 81.143, center_y = 95.143, center_z = 93.644) and box grid parameters (size_x = 50, size_y = 60, size_z = 50) of the P50 receptor, the active pocket sites to which the small-molecule ligands might bind were covered. AutoDock Vina 1.1.2 was used to perform molecular docking between P50 receptor and Pra ligand small molecules. The hydrogen bond interaction between the receptor and the small molecules was visualized in 3D via PyMOL, while their hydrophobic interaction was visualized in 2D using Ligplus software.

Recombinant P50 receptor (15 μg) and Biotin-labeled Pra (Biotin-Pra) were bound, and the recombinant protein G magnetic beads were incubated with P50 antibody. After washing with Tris buffer, P50 was determined as per the aforementioned Western-Blot procedure, and biotin detection was carried out with the HRP-conjugated antibiotin antibody (CST, MA, USA).

### Statistical methods

SPSS 20.0 was used to perform statistical analyses, and all measurement data were expressed as ($\bar{x}\;\pm \text{ s}$). One-way ANOVA was used for comparison among multiple groups, while SNK test was employed to make pairwise comparisons. The differences were considered statistically significant when *P* < 0.05.

### Data availability statement

The data that support the findings of this study are available from the corresponding author upon reasonable request.

## RESULTS

### Par inhibits the metastatic, invasive and glycolytic abilities of HCC cells

**Figure 1:**
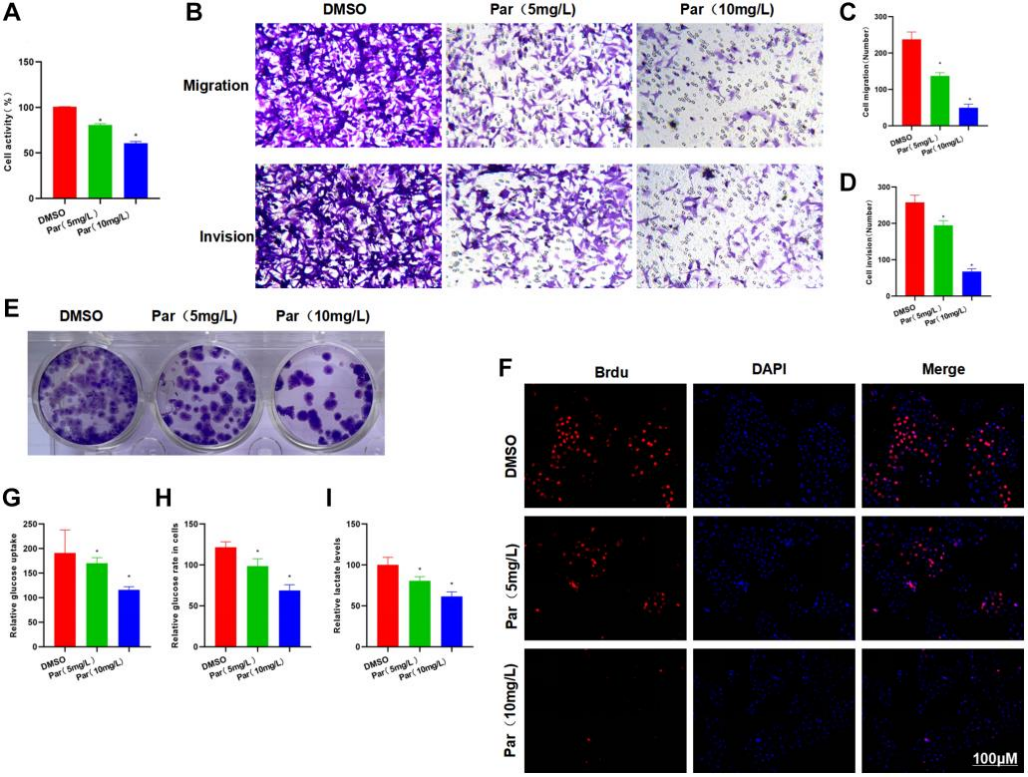
**Par inhibits the metastatic, invasive and glycolytic abilities of H22 cells.** (**A**) Cell viability assay revealed that Par could inhibit the viability of H22 cells in a dose-dependent manner. ^*^P < 0.05 vs. DMSO group. (**B**–**D**) Transwell assay found that Par could inhibit the metastasis and invasion of H22 cells, as manifested by significantly reduced numbers of metastatic and invasive cells. ^*^P < 0.05 vs. DMSO group. (**E**) According to the results of clone formation assay, Par inhibited the cell clone formation, leading to reduced number of colonies as compared to the DMSO group. (**F**) After Brdu staining, many positive cells were present in the DMSO group, indicating that H22 cells were in the proliferative phase. Par group exhibited significantly lower number of positive cells than the DMSO group. Par could inhibit the H22 cell proliferation. (**G**–**I**) Par suppressed the glycolytic ability of cells, inhibited the cellular absorption of glucose, lowered the intracellular glucose level and decreased the lactate release in a dose-dependent manner. ^*^P < 0.05 vs. DMSO group.

Our treatment at high and low doses found that Par could inhibit the viability of H22 cells. The cell viability was significantly lower than that of the DMSO group. Meanwhile, the cells in the high-dose group were less viable than those in the low-dose group (Figure 1A). Transwell assay revealed that Par could inhibit the metastasis and invasion of H22 cells in a dose-dependent manner (Figure 1B–1D). The results of clone formation assay also showed the ability of Par to inhibit the H22 clone formation, as manifested by the significantly reduced number of clones, which was lower than that in the DMSO group (Figure 1E). According to Brdu staining results, there were many positive cells in the DMSO group, indicating that H22 cells were in the proliferative phase. Par group exhibited significantly decreased number of positive cells as compared to the DMSO group. Par could inhibit the proliferation of H22 cells (Figure 1F). As suggested by glycolysis assay results, Par could inhibit the cellular absorption of glucose, lower the intracellular glucose level and decrease the lactate release (Figure 1G–1I).

**Figure 2:**
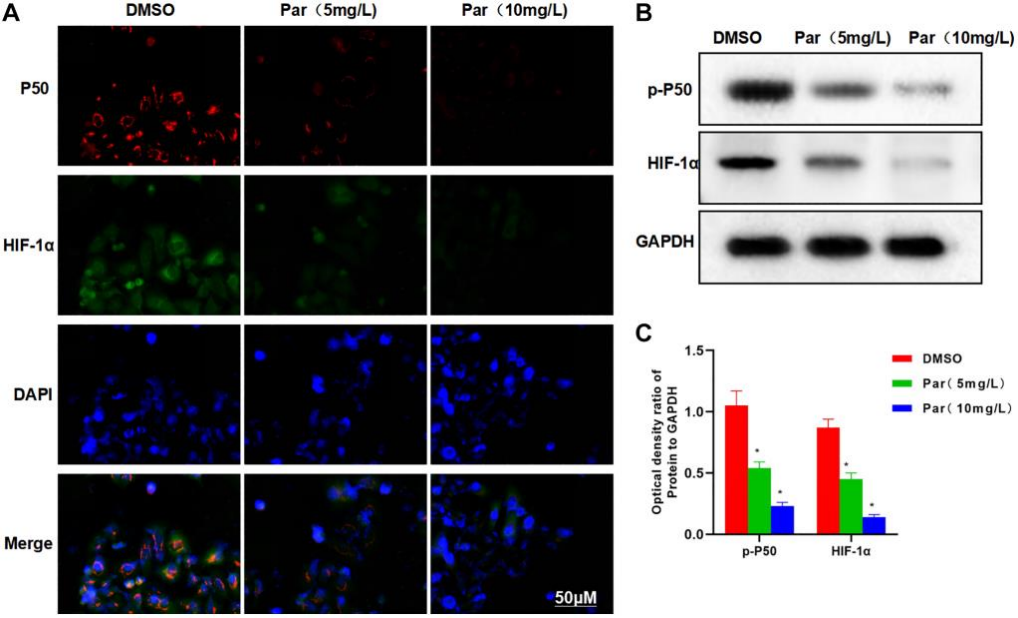
**Par inhibits the metastatic, invasive and glycolytic abilities of H22 cells.** (**A**) IFA revealed high expression levels of P50 and HIF-1α in H22 cells, with strong fluorescence intensities. Par could suppress the P50 and HIF-1α expressions, as manifested by the significantly weakened fluorescence intensity in the Par group. (**B**, **C**) Protein assay demonstrated that Par inhibited the p-P50 and HIF-1α expressions in a dose-dependent manner. ^*^P < 0.05 vs. DMSO group.

IFA revealed high expression levels of P50 and HIF-1α in H22 cells, with strong fluorescence intensities. Par could suppress the P50 and HIF-1α expressions, as manifested by the significantly weakened fluorescence intensity in the Par group than in the DMSO group (Figure 2A). Protein assay results also demonstrated that Par inhibited the expressions of p-P50 and HIF-1α (Figure 2B, 2C).

### Silencing P50 inhibits the metastatic, invasive and glycolytic abilities of HCC cells

**Figure 3:**
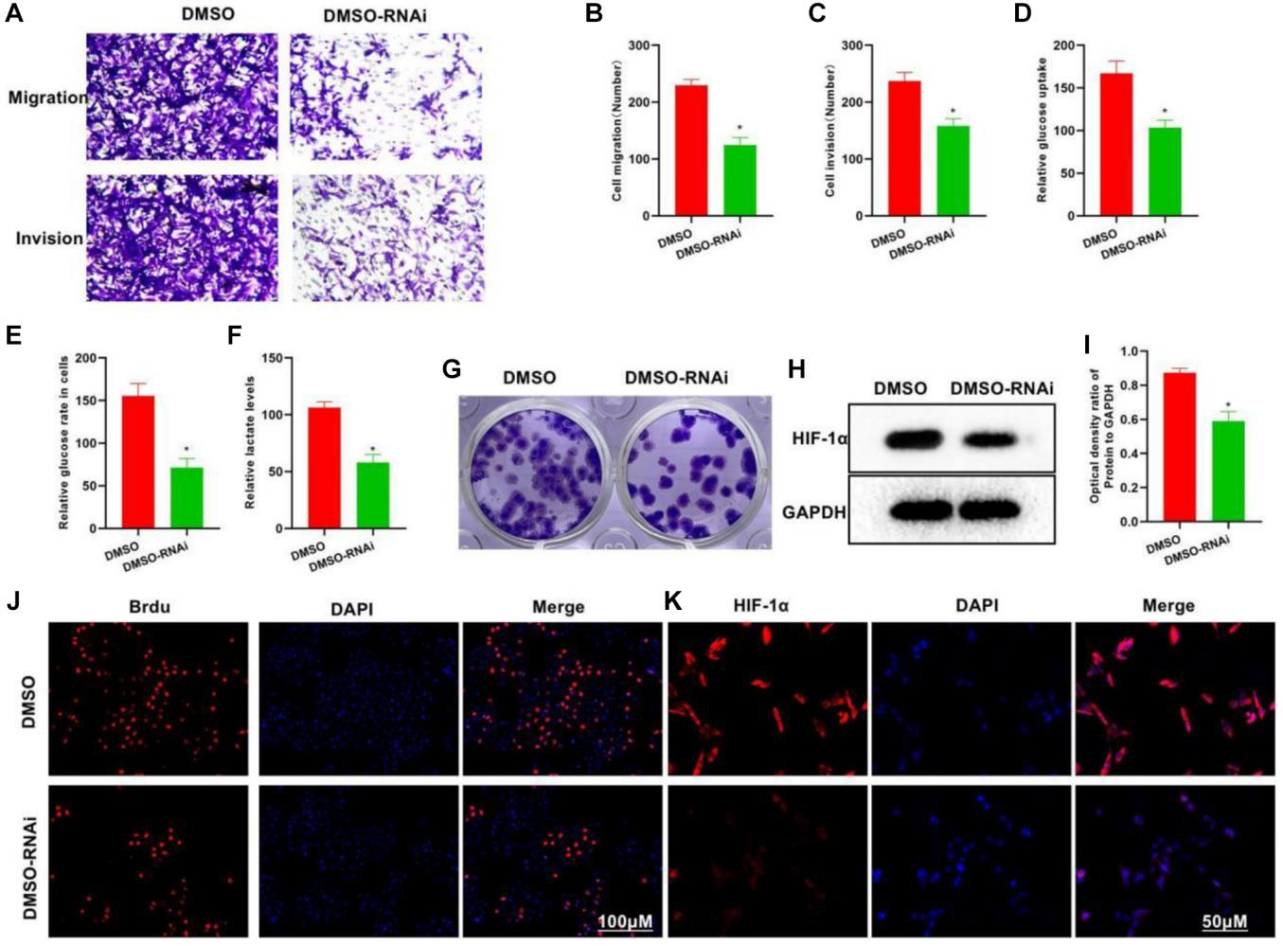
**Silencing P50 inhibits the metastatic, invasive and glycolytic abilities of H22 cells.** (**A**–**C**) In the DMSO-RNAi group, the invasive and metastatic abilities of cells weakened drastically, showing lower numbers of invasive and metastatic cells than in the DMSO group. ^*^P < 0.05 vs. DMSO group. (**D**–**F**) Compared to the DMSO group, the DMSO-RNAi group exhibited significantly reduced glucose uptake, intracellular glucose level and lactate expression. ^*^P < 0.05 vs. DMSO group. (**G**) Reduced number of clones formed was noted in the DMSO-RNAi group. (**H**, **I**) According to both HIF-1α IFA and Western-Blot results, the expression of HIF-1α could be suppressed after silencing P50. ^*^P < 0.05 vs. DMSO group. (**J**, **K**) The number of Brdu-positive cells in the DMSO-RNAi group was less than that in the DMSO group, suggesting inhibition of the cell proliferation.

To explore the role of P50 in glycolysis, we silenced P50. In the DMSO-RNAi group, the invasive and metastatic abilities of cells weakened drastically, showing lower numbers of invasive and metastatic cells than the DMSO group (Figure 3A–3C). Meanwhile, the DMSO-RNAi group exhibited significantly reduced glucose uptake, intracellular glucose level and lactate expression as compared to the DMSO group (Figure 3D–3F). The clone formation assay also revealed decreased number of clones in the DMSO-RNAi group (Figure 3G). Proliferation assay found less number of Brdu-positive cells in the DMSO-RNAi group than in the DMSO group, suggesting inhibition of the cell proliferative potential (Figure 3H). Both IFA and protein assay revealed that after P50 silencing, the expression of HIF-1α could be suppressed (Figure 3J, 3K and 3I).

### Silencing P50 inhibits the effect of par

**Figure 4:**
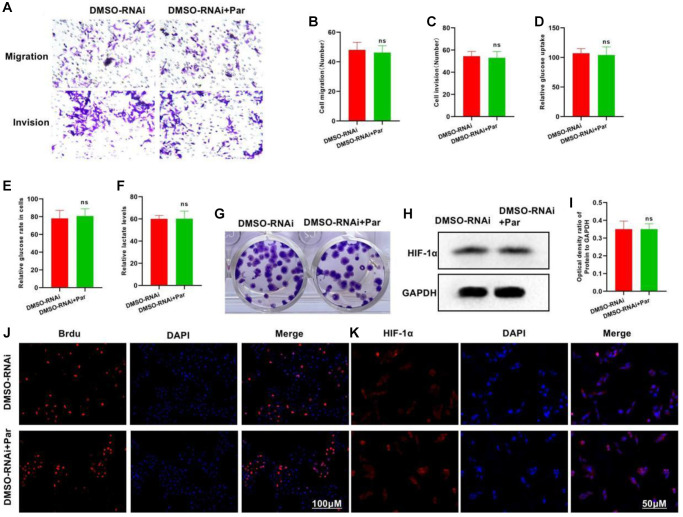
**Silencing P50 inhibits the effect of Par.** (**A**–**C**) DMSO-RNAi+Par group differed insignificantly from DMSO-RNAi group regarding the numbers of invasive and metastatic cells. ^ns^*P* > 0.05 vs. DMSO-RNAi group. (**D**–**F**) In the glycolysis assay, the glucose uptake, intracellular glucose level and lactate expression of the DMSO-RNAi+Par group differed insignificantly from those of the DMSO-RNAi group. ^ns^*P* > 0.05 vs. DMSO-RNAi group. (**G**) Insignificant difference in the number of plate clones was noted between the DMSO-RNAi+Par and the DMSO-RNAi groups. (**H**, **I**) After P50 silencing, Par exerted an insignificant effect on the HIF-1α expression. ^ns^*P* > 0.05 vs. DMSO-RNAi group. (**J**, **K**) Difference in the Brdu-positive cell count was insignificant between the DMSO-RNAi+Par and the DMSO-RNAi groups.

We treated the P50-silenced cells with Par, and found that Par was incapable of further inhibiting the cellular metastasis or invasion, nor could it inhibit glycolysis. In the Transwell assay, insignificant differences were found in the number of invasive or metastatic cells between the DMSO-RNAi+Par and the DMSO-RNAi groups (Figure 4A–4C). In the glycolysis assay, the glucose uptake, intracellular glucose level and lactate expression of the DMSO-RNAi+Par group differed insignificantly from those of the DMSO-RNAi group (Figure 4D–4F). Regarding the number of plate clones, the inter-group difference was also insignificant (Figure 4G). In Brdu staining, the positive cell count in the DMSO-RNAi+Par group differed insignificantly from that in the DMSO-RNA group (Figure 4H). Both IFA and protein assay revealed that after P50 silencing, Par exerted an insignificant effect on the HIF-1α expression (Figure 4J, 4K and 4I).

### Par inhibits the tumor growth

**Figure 5:**
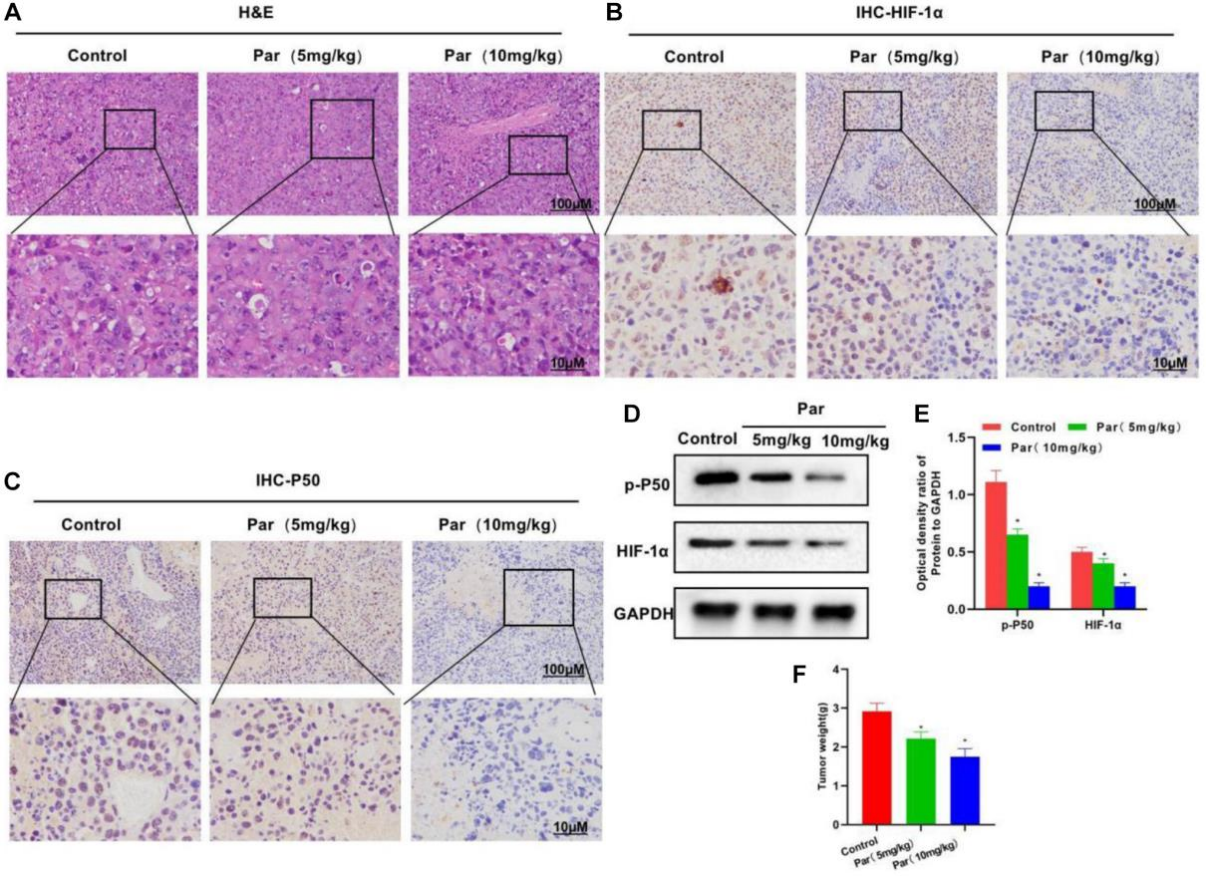
**Par inhibits the tumor growth.** (**A**) H&E staining revealed an impotent killing activity of Par against tumor tissues, without producing obvious tissue inflammation or evident toxic effect. (**B**–**E**) In the IHC staining and Western-Blot assays, Par inhibited the tissue expressions of P50 and HIF-1α in a dose-dependent manner. ^*^P < 0.05 vs. Control group. (**F**) In the tumor mass detection, Par could inhibit the growth of tumor, significantly reducing the tumor mass. ^*^P < 0.05 vs. Control group.

After establishing the tumor-bearing mouse model, we intervened it with Par. H&E staining revealed that Par had an impotent killing activity against tumor tissues, without obvious tissue inflammatory reaction or evident toxic effect, which differed little from the Control group (Figure 5A). After IHC staining, P50 and HIF-1α were found highly expressed in the Control group. Par inhibited the tissue expressions of P50 and HIF-1α in a dose-dependent manner. A positive correlation was present between the P50 and HIF-1α expressions (Figure 5B, 5C). In the tumor mass detection, Par was found to inhibit the growth of tumor, as manifested by significantly decreased tumor mass than that in the Control group (Figure 5D). Protein assay also revealed that Par inhibited the expressions of p-P50 and HIF-1α in a dose-dependent manner (Figure 5E, 5F).

### Identification of par–P50 binding relationship

**Figure 6:**
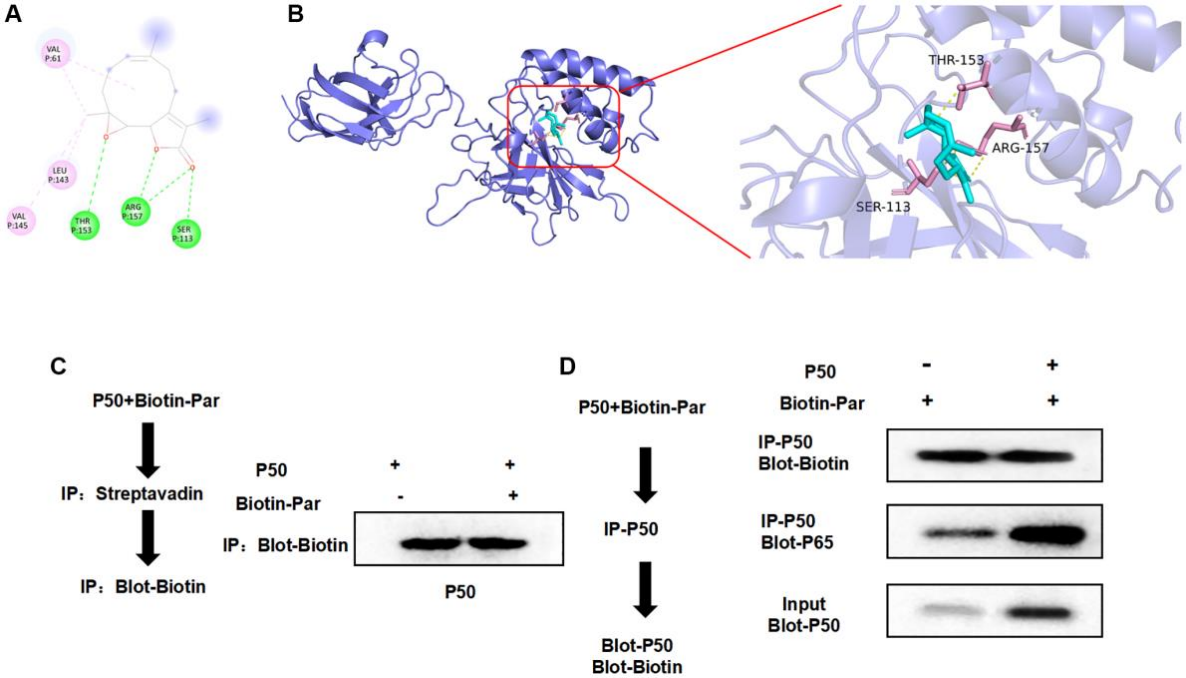
**Targeted binding relationship between Par and P50.** (**A**, **B**) According to the 3D and 2D images after Par–P50 binding, Par showed hydrogen bonding with THR, ARG and SER, and showed hydrophobic alkyl bonding with VAL and LEU. (**C**, **D**) Pull-down experiment revealed the presence of targeted binding between Par and P50, which agreed with the small molecule–protein docking results.

Through small molecule–protein docking, we found that the binding energy of Par and P50 was −5.9 Kcal/mol. Par showed hydrogen bonding with THR, ARG and SER, as well as alkyl bonding with VAL and LEU. Small molecules bound near the phosphorylation sites (Figure 6A, 6B). In the pull-down experiment, we found that P50 could bind to Par (Figure 6C, 6D).

## DISCUSSION

Glycolysis is the main energy source for tumor cells. Hypoxia, as a common feature of solid tumors, plays a pivotal role in tumor progression [8]. Otto Warburg et al. found that many tumor cells produce excessive lactate even under normoxia, which is known as pseudohypoxia [9]. Enhanced glycolytic activity has been shown to be closely associated with elevated level of HIF-1α. Subsequent research has also confirmed that the HIF-1α family is the primary mediator of change in energy metabolism of tumor cells from aerobic phosphorylation to aerobic glycolysis [10]. Mediated by HIF-1α, tumor cells up-regulate a range of genes related to the glycolytic metabolism, angiogenesis, tumor cell survival and erythropoiesis [11], including vascular endothelial growth factor (VEGF) [12], erythrogenin (EPO), glucose transporter (GLUT) [13] and some other glycolytic enzymes, ultimately promoting the Warburg effect. Thus, HIF-1α is one of the major promoters of the Warburg effect, which is also an important therapeutic target for abnormal glucose metabolism in tumors [14]. The expression of HIF-1α is regulated by NF-κB signaling. Hypoxia can also activate the intracellular NF-κB pathway [15]. NF-κB is a series of dimeric transcription factors composed of different subunits p65 (RelA), RelB, c-Rel, NF-κB1 (p105/p50) and NF-κB2 (p100/p52), which plays an important role in regulating cell survival and immune response [16]. Activated NF-κB in HCC cells has been found to promote the expressions of inflammatory cytokines (e.g., IL-6, IL-1β), which creates a cancer-promoting inflammatory microenvironment and is involved in the carcinogenesis and progression of HCC [17]. The interaction between HIF-1α and NF-κB may play a crucial role in maintaining the hypoxia response of cells [18]. According to previous reports, the NF-κB subunits p50 and p65 can stimulate the HIF-1α transcription [19]. Meanwhile, HIF-1α also participates in regulating the NF-κB activation. It can be said that HIF-1α and NF-κB are mutually interactive.

Par is a sesquiterpene lactone isolated from *Tsoongiodendron odorum* Chun. and *Chrysanthemum parthenium* [20]. Domestic and foreign research has shown a strong *in-vitro* anti-tumor activity of Par, which has an anti-proliferative effect on multiple tumors, including colon cancer, liver cancer, cholangiocarcinoma, acute/chronic leukemia and multiple myeloma [21]. According to the latest research, Par can preferentially act on acute myelogenous leukemia (AML) stem/progenitor cells, and induce potent apoptosis of primary human AML cells and acute-phase cells of chronic myelogenous leukemia (CML) without affecting normal hematopoietic cells, which is more specific to leukemia cells than cytarabine [22]. In our study, we explored the effect and role of Par on the glucose metabolism reprogramming using a HCC model. H22 is a common invasive cell line. Par can inhibit the ability of H22 cells to metastasize and invade while suppressing relevant clone formation. This suggests certain inhibitory effect of Par on the proliferation and growth of H22 cells. Our Brdu results showed that Par reduced the positive cell count, which also corroborated our assumption. Detection of glycolytic ability found that Par could inhibit the cellular absorption of glucose, lower the intracellular level of glucose and reduce the lactate production [23], implying the association of Par’s anti-H22 effect with the glucose metabolism reprogramming. According to our P50 and HIF-1α measurements, Par inhibited the expressions of P50 and HIF-1α. To clarify the role of P50, which is an important protein of NF-κB and an upstream signal of HIF-1α [24], we silenced P50 in H22 cells and found that P50 silencing could inhibit the cellular metastasis and invasion, and suppress the glycolysis. RNAi treatment inhibited the glucose absorption and lactate production by cells, as well as the HIF-1α level. Hence, we confirm that P50 can regulate the expression of HIF-1α by adjusting cell glucose metabolism. Through small molecule–protein docking, we found that Par exerted its effect primarily by binding to P50. The Pull-down experiment also verified this point. Our Par treatment of RNAi-treated H22 cells showed that Par was no longer effective after P50 inhibition, with insignificantly down-regulated abilities of cellular metastasis and invasion and no change in glycolytic ability. This further proves that Par exerts its effect by targeting P50. In animal model, Par could inhibit tumor growth, as manifested by significantly smaller tumor mass than the Control group. Meanwhile, Par could also inhibit the cellular expressions of P50 and HIF-1α, showing agreement with the cell experiment results. All the above effects were dose-dependent. In H&E staining, Par showed insignificant toxic effect on tissues, which might be attributable to the dose. Thus, we are more certain that metabolic reprogramming is the primary anti-HCC mechanism of Par.

## CONCLUSION

This study discovers that Par can target P50 to inhibit the expression of downstream HIF-1α, a key factor in tumor glycolysis. Hence, Par regulates the glycolysis of HCC cells by inhibiting NF-κB-HIF-1α pathway, ultimately inhibiting the HCC cell metastasis and invasion. P50 has potential as a novel therapeutic target for HCC, while Par can be a candidate drug for HCC treatment.
